# 

**Published:** 1884-10

**Authors:** 


### BOOKS AND PAMPHLETS RECEIVED.

Disease in Cattle, Kansas and Illinois. Report of James Law, Chairman of the Treasury Cattle Commission. Washington.

Report of the U. S. Treasury Cattle Commission for the Year 1883. Washington.

Annual Report of the Board of Regents of the Smithsonian Institute for the Year 1882. Washington, 1884.

Annual Announcement of the New York College of Veterinary Surgeons and School of Comparative Medicine, Session of 1884–85.

ANNUAL ANNOUNCEMENT OF THE COLUMBIA VETERINARY COLLEGE AND SCHOOL OF COMPARATIVE MEDICINE, SESSION OF 1884-85.

THE DIRECT INFLUENCE OF GRADUAL VARIATIONS OF TEMPERATURE UPON THE RATE OF BEAT OF THE DOG'S HEART By H. Newell Martin, M.A., M.D., D.Sc., Professor in the Johns Hopkins University. From the Philosophical Transactions of the Royal Society, Part II, 1883.

RELATION OF VETERINARY MEDICINE TO AGRICULTURE. An Address delivered by Thomas Walley, M.R.C.V.S., Principal Royal Veterinary College, Edinburgh, before the Scottish Metropolitan Veterinary Medical Association, July 24, 1884.

CATALOGUE OF THE NATIONAL UNIVERSITY, WASHINGTON, 1884-85.

JAHRESBERICHT DER KONIGLICHEN THIERARZNEISCHULE ZU HANOVER. Herausgegeben von den Lehrer-Collegium redigirt von den Director· Dr. Dammann. Sechszehnter Berecbt, 1883-84. Hanover, 1884.

JOHNS HOPKINS UNIVERSITY CIRCULARS.

ANNALS ET BULLETIN DE LA SOCIÉTIÉ DE MEDICINE DE GAND.

JOURNALS.—La Clinica Veterinaria, Milano. The Veterinarian, London; The Veterinary Journal, London; Der Zoologische Garten, Frankfort; Schweizerisches Archiv fur Thierheilkunde, Zurich; Archiv fur Wissenschaftliche und Praktische Thierheilkunde, Berlin; Wochenschrift fur Thierheilkunde und Viehzucht, Augsburg; Repertorium der Thierheilkunde, Stuttgart; Journal de la Société contre L'Abus du Tabac, Paris; Giornale di Anatomia, Fisiologia e Patologia, degli Animali, Pisa; Il Medico Veterinario, Torino; El Ensayo Medico, Caracas; La Cronica Medica, Valencia; Kansas City Review of Science and Industry; Chicago Medical Journal and Examiner; The College and Clinical Record, Philadelphia; New England Medical Monthly, Sandy Hook; Chicago Medical Review; Virginia Medical Monthly, Richmond; Nashville Journal of Medicine and Surgery; The Southern Clinic, Richmond; The Western Medical Reporter, Chicago; Journal of Cutaneous and Venereal Diseases, New York; Denver Medical Times; The St. Joseph Medical Herald; The Weekly Medical Review, St. Louis; The Blacksmith and Wheelwright, New York; National Live Stock Journal, Chicago; The Breeder's Gazette, Chicago; Cultivator and Country Gentleman, Albany; Weekly Drover's Journal, Chicago; The Chicago Tribune; United States Veterinary Journal, Chicago; Horseshoer and Hardware Journal, Chicago; The Polyclinic, Philadelphia; Medical World, Philadelphia; American Veterinary Review, New York; Atlantic Journal of Medicine, Richmond; Indiana Farmer, Indianapolis; Texas Courier Record, Fort Worth; The Jersey Bulletin, Indianapolis; American Journal of Ophthalmotology, St. Louis; Pacific Medical and Surgical Journal and Western Lancet, San Francisco.; New York Medical Journal; The Quarterly Journal of Veterinary Science in India.

